# The Role of AKT/mTOR Pathway in Stress Response to UV-Irradiation: Implication in Skin Carcinogenesis by Regulation of Apoptosis, Autophagy and Senescence

**DOI:** 10.3390/ijms140815260

**Published:** 2013-07-24

**Authors:** Elwira Strozyk, Dagmar Kulms

**Affiliations:** Experimental Dermatology, Department of Dermatology, TU Dresden, 01307 Dresden, Germany; E-Mail: dagmar.kulms@uniklinikum-dresden.de

**Keywords:** UV, DNA damage, p53, AKT, mTOR, cell death, apoptosis, autophagy, senescence, heat shock

## Abstract

Induction of DNA damage by UVB and UVA radiation may generate mutations and genomic instability leading to carcinogenesis. Therefore, skin cells being repeatedly exposed to ultraviolet (UV) light have acquired multilayered protective mechanisms to avoid malignant transformation. Besides extensive DNA repair mechanisms, the damaged skin cells can be eliminated by induction of apoptosis, which is mediated through the action of tumor suppressor p53. In order to prevent the excessive loss of skin cells and to maintain the skin barrier function, apoptotic pathways are counteracted by anti-apoptotic signaling including the AKT/mTOR pathway. However, AKT/mTOR not only prevents cell death, but is also active in cell cycle transition and hyper-proliferation, thereby also counteracting p53. In turn, AKT/mTOR is tuned down by the negative regulators being controlled by the p53. This inhibition of AKT/mTOR, in combination with transactivation of damage-regulated autophagy modulators, guides the p53-mediated elimination of damaged cellular components by autophagic clearance. Alternatively, p53 irreversibly blocks cell cycle progression to prevent AKT/mTOR-driven proliferation, thereby inducing premature senescence. Conclusively, AKT/mTOR via an extensive cross talk with p53 influences the UV response in the skin with no black and white scenario deciding over death or survival.

## 1. Introduction

**Figure 1:**
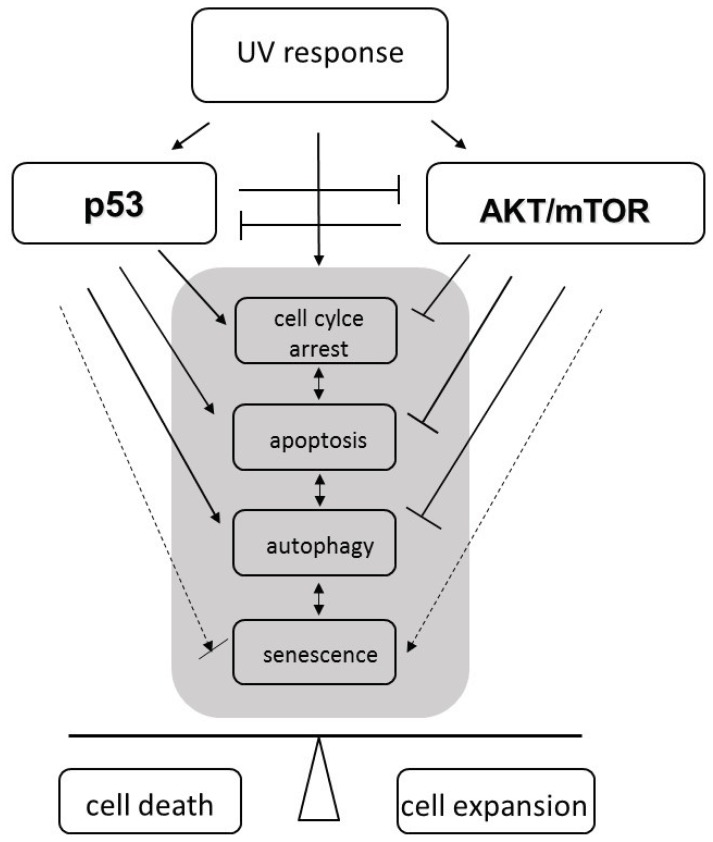
The role of p53 and AKT/mTOR in cellular responses to ultraviolet (UV) radiation. UV activates both, p53 and AKT/mTOR signaling pathways. An intact p53 response in irradiated cells leads to cell cycle arrest to enable damage repair and eventually to induce apoptotic cell death when the damage is too severe and/or repair remains incomplete. Cell cycle arrest and apoptosis are negatively regulated by AKT/mTOR activity. Thus, AKT/mTOR can enforce proliferation. It also prevents autophagy, a mechanism to recycle damaged proteins or organelles that remain under the control of p53. So far, AKT/mTOR can counteract the activity of p53 in response to UV irradiation and *vice versa*. At last, p53 in concert with AKT/mTOR signaling can drive cells to premature senescence, an irreversible cell-cycle arrest that counteracts oncogenic transformation. Shifting the balance between p53 and AKT/mTOR signaling can determine between either cell death or survival and clonal expansion of irradiated cells.

An increasing exposure to ultraviolet light (UV) from either sun-emitted radiation or artificial sources (e.g., in medical applications or wellness facilities), represent one of the major hazards affecting human health by skin carcinogenesis [1]. The process of malignant transformation in the skin is associated with UV-induced DNA damage, which causes mutations when left unrepaired. Genetic defects in oncogenes and tumor suppressors provoke disturbance of the cell cycle control and proliferation, which further leads to the uncontrolled expansion of altered cells. Protection from the deleterious alterations and expansion of cancer-prone cell clones relies on the elimination of damaged cells via UV-induced apoptosis. Both processes, cell cycle control and apoptosis, represent a cellular DNA damage response converging on the tumor suppressor p53. As a result of chronic irradiation, excessive cell death may lead to permanent tissue damage. Thus, in order to maintain the integrity of the skin barrier, UV responses additionally include mechanisms by which cells can survive under stress conditions. Among these, AKT/mTOR signaling reciprocally interacting with p53 emerges as a potential life/death regulator of irradiated skin cells (Figure 1). Upon activation by UV irradiation AKT/mTOR not only inhibits apoptosis, but also forces cell cycle transition and counteracts stress-induced autophagy. Consequently, unbalanced AKT/mTOR signaling may eventually lead to hyper-proliferation and contribute to malignant transformation. Although the oncogenic potential of AKT/mTOR may also drive cells into senescence, the involvement of this process in the overall UV response remains unclear. Altogether, UV-induced modifications in the activity of signaling factors involved in anti- and pro-survival pathways may significantly alter cellular stress responses that interfere with UV-induced cell death. As a consequence, the balance is shifted from cell death to malignant transformation and to clonal expansion of UV-damaged cells. Moreover, UV exposure is usually accompanied by generation of heat, causing adaptive stress response utilizing heat shock proteins. Thus, the impact of heat on UV-induced cell death has to be considered when evaluating beneficial and/or adverse effects of UV radiation. This review is intended to recapitulate the recent knowledge of how the complex intracellular signaling network comprising apoptosis, autophagy, senescence and heat shock responses may orchestrate the effects of UVB and UVA in the context of skin cancer development. Special emphasis is placed on the AKT/mTOR driven pathways, which have been shown to play a decisive role in - malignant transformation [2].

## 2. Mutagenicity of UV Radiation as a Prerequisite for Skin Cancer Development

The decrease of the stratospheric ozone layer plus indoor applications of UV light increases the exposure of human skin to the hazardous effects of UVB and UVA radiation [1]. Due to its wavelength (280–320 nm) UVB is known to be the most potent mutagenic component causing direct damage to cellular DNA as well as production of reactive oxygen species (ROS) in the epidermis, dermis and the corneal epithelium [3–5]. Major photolesions induced by UVB comprise cyclobutane pyrimidine dimers (CPDs) and pyrimidine-pyrimidone (6–4) photoproducts ((6–4)PPs) [3]. Since removal of (6–4)PP by specific repair machinery of nuclear excision (NER) is more effective than of CPDs, the mutagenic potential of CPDs is superior and is responsible for 80% of UVB-induced mutations [3,6]. CPDs are commonly induced between two adjacent pyrimidines, thymines (T) and/or cytosines (C). TC to TT or CC to TT transitions turned out to be the major mutagenic events during skin tumor development and are referred to as UV fingerprint mutations [3,7]. Genotoxicity of UVA (320–390 nm), which penetrates deeply into the subcutaneous tissue and reaches retinal cells of the eye, has long been believed to be dependent mainly on indirect mechanisms involving generation of ROS. These cause transient DNA breakage and/or induction of oxidative modifications of pyrimidines such as thymine glycol, and purines such as 8-oxo-7,8-dihydro-2′-deoxyguanosine (8-oxoG), the latter anticipated to cause G to T transversions [5,8,9]. However, recent data indicated that UVA induces CPDs as potently as UVB whereas their removal is less efficient than those induced by UVB. Thus, UVA was recently acknowledged to have an even higher mutagenic potential than UVB [4,10–13]. Since UVA contributes significantly to malignant transformation of exposed cells, the characteristic mutational repertoire (UV-fingerprint) cannot exclusively be ascribed to one type of UV radiation. Importantly, UV-induced photolesions not only predispose cells to mutational changes but also contribute to genomic instability due to defective replication and transcription. UV-induced photolesions distort DNA replication forks, thereby introducing double strand breaks (DSBs), which are generally sensed and processed via homologous recombination repair (HRR) and non-homologous end joining (NHEJ) [14].

Indeed both, UVB and UVA represent carcinogens for non-melanoma skin cancer, including squamous cell carcinoma (SCC) and basal cell carcinoma (BCC) and are emerging as causative factors in malignant melanoma (MM) formation [3,9,13,15]. All of these cancers are characterized by a high frequency of *TP53* mutations with UV-fingerprint, indicating that p53 plays a major role in preventing UV-induced carcinogenesis [3]. Recently, a new mechanism of UVA-mediated melanomagenesis has been proposed. It is based on the finding that melanin produced by melanocytes, which is distributed to the surrounding keratinocytes to protect them against the hazardous UV effects, can also act as a photosensitizer. Thereby melanin contributes to oxidative DNA damage in melanocytes and may consequently play a critical role in UVA-induced melanoma formation [16–18].

Undoubtedly, UV radiation represents one of the major etiological factors responsible for the development of skin cancer. The question remains how the complex cellular machinery for DNA damage recognition and repair coupled to cell cycle arrest and/or execution of cell death can be suspended? Additionally to causing genomic DNA damage, the multifactorial effects of UV also include damage in mitochondrial DNA [19], modulation of signal transduction pathways by ROS formation, changes in the activity of surface receptors and a variety of kinases, phosphatases and transcription factors [20]. Taken together, carcinogenesis occurs via mutagenic DNA modifications in susceptible cells due to insufficient repair and pathological enforcement of survival pathways and/or deregulation of check point control. In this context, oncogenic AKT signaling pathway activated in response to UV is emerging to be an important player due to its multiple targets involved in cell survival and cell death [2,21–31].

## 3. AKT Potently Mediates Oncogenic Signaling

**Figure 2:**
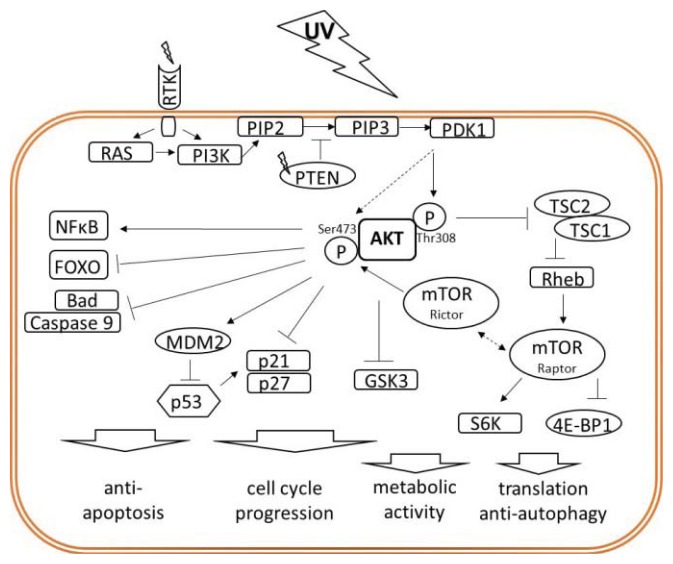
Oncogenic role of the AKT signaling pathway. UV-triggered RTK or functional inactivation of PTEN leads to activation of AKT. Activated AKT signals to activate the anti-apoptotic transcription factor NFκB or inhibits pro-apoptotic molecules such as the transcription factor FOXO, Bad, or caspase 9. By activation of the p53 inhibitor MDM2, AKT antagonizes p53-mediated responses: *i.e.*, cell cycle arrest and apoptosis induction. AKT forces cell cycle progression by blocking cell cycle control proteins p21 and p27, and via inhibition of GSK3 kinase stabilizes cyclins and drives cellular metabolism. AKT-mediated inhibition of TSC2 leads to activation of mTOR/Raptor (mTORC1), which controls protein synthesis and autophagy. Activated by a currently unknown mechanism mTOR/Rictor (mTORC2) mediates critical phosphorylation and activation of AKT.

Serine/threonine protein kinase B (AKT) becomes activated downstream of the receptor tyrosine kinase (RTK)-activated PI3K by phosphorylation at Thr308 [26]. AKT gains its full kinase activity upon additional phosphorylation of Ser473 by the mTOR complex 2 (mTORC2) [26,32]. Activation of AKT is negatively regulated by the PTEN (Phosphatase and Tensin homolog deleted from chromosome ten) phosphatase, a tumor suppressor frequently mutated in different tumors, including melanoma [31,33]. In response to UV radiation AKT can be activated in an autocrine fashion or by ligand-independent but ROS-dependent triggering of growth factor receptors bearing RTK activity [22,23,34–37], as well as by functional loss of PTEN [38,39]. Therefore, AKT-mediated activation of MDM2, a negative regulator of p53, can have a significant impact on UV stress responses by preventing p53-mediated cell cycle arrest and apoptosis induction (Figure 2) [40,41]. In contrast, AKT regulates other target proteins, which may force cell cycle progression and consequently proliferation. Thus, AKT prevents nuclear translocation of cell cycle inhibitors p21^Cip1/WAF1^ and p27^Kip1^, and by phosphorylation and inhibition of the glycogen synthase kinase 3 (GSK3) stabilizes cyclins and increases metabolic activity [2,26,27]. Moreover, by inhibition of tuberous sclerosis protein 2 (TSC2), AKT activates mammalian target of rapamycin (mTOR) [2,42]. Within the rapamycin sensitive complex 1 (mTORC1) containing also Raptor, mTOR promotes cell growth and proliferation by regulating protein synthesis. In this context, mTORC1 fosters translation via phosphorylation of p70/p85 S6 kinase (S6K) and eukaryotic initiation factor 4E (eIF-4E) binding protein-1 (4E-BP1) [43]. During starvation, mTOR becomes inactivated by the AMP-activated protein kinase (AMPK) pathway, what contributes to catabolic processes, growth suppression and induction of autophagy [44]. AMPK has also been reported to counteract AKT-mediated activation of mTOR in response to UV radiation and together with activation of p53 accounts for the pro-apoptotic pathways downstream from ROS-activated growth factors receptors [37,45]. The mechanism by which the rapamycin insensitive mTORC2 complex, harbouring the specific subunit Rictor,becomes activated remains elusive. However, it plays a fundamental role in AKT signaling since it controls phosphorylation of AKT at Ser473 and therefore its full kinase activity [32]. Thus, mTOR is a target of AKT but *vice versa*, mTOR also targets AKT forming a signaling axis referred to as the AKT/mTOR pathway. Besides its crucial role in pro-survival signaling, AKT actively inhibits apoptosis. Apart from p53 inhibition, AKT negatively regulates the pro-apoptotic forkhead transcription factors (FOXO) while activating the anti-apoptotic nuclear factor kappa B (NFκB) [29–31]. Anti-apoptotic function of AKT additionally comprises inactivating phosphorylation of pro-apoptotic proteins Bad and caspase 9. Altogether, these data provide strong evidence for a multilayer anti-apoptotic/oncogenic profile being offered by activated AKT [2,21,28].

## 4. Interplay Between AKT and p53 Modulates UV-Induced DNA Damage Responses

The decision whether apoptosis is induced or not is made during a temporary cell cycle arrest in which the balance between numerous kinases senses the intensity of DNA damage and ponder cell cycle progression against apoptosis induction. The major kinases involved are ATM (ataxia telangiectasia mutated), ATR (ataxia telangiectasia and Rad3-related) and DNA-PK (DNA-dependent protein kinase). While ATM and DNA-PK mainly become activated in response to ionizing radiation, ATR predominantly responds to UV radiation, causing phosphorylation of histone H2AX, phosphorylation of check point kinases (Chk1/2), and phosphorylation of p53 at Ser15 and Ser20 [14,46,47]. Modified histone H2AX serves as a binding site for repair enzymes and checkpoint proteins while activated and stabilized p53 triggers transient cell cycle arrest in G1 via up-regulation of p21 and contributes to effective G2/M transition control [14,48,49].

Proper DNA repair as well as activation of checkpoint proteins also depends on the activation status of AKT. Although some data indicate that AKT may support UV-induced DSBs repair [50,51] conversely, high AKT activity can suppress ATR/Chk1 signaling and HRR via direct phosphorylation of Chk1 or, indirectly, by inhibiting recruitment of DSB resection factors [27]. If repair during cell cycle arrest is insufficient, p53 commits the cell to apoptotic cell death. Generally, activated p53 regulates the intrinsic apoptotic pathway based on mitochondrial dysfunction resulting from a misbalance between pro-(Bax, Bak, Bad, Bid, Bim) and anti-apoptotic (Bcl-2, Bcl-x_L_, Bcl-w, Mcl-1) members of the Bcl-2 family. Under normal physiological conditions, anti-apoptotic members sequester pro-apoptotic counterparts via binding to homologous BH3 domains thereby guaranteeing mitochondrial membrane integrity [52]. Upon UV-induced DNA damage, p53-triggered up-regulation of genes coding for pro-apoptotic Bax, Bak, PUMA and Noxa proteins in concert with trans-repression of anti-apoptotic Bcl-2, Bcl-x_L_ [53,54], or direct binding of p53 to the mitochondria [55] leads to loss of the mitochondrial membrane potential. Consequently, the release of cytochrome c into the cytoplasm causes—together with Apaf-1 and pro-caspase-9—formation of the apoptosome leading to ATP-dependent caspase-9 maturation. This finally results in proteolytic activation of downstream effector caspases 3, 6, and 7, which ultimately cleave cellular death substrates to execute apoptosis [56].

**Figure 3:**
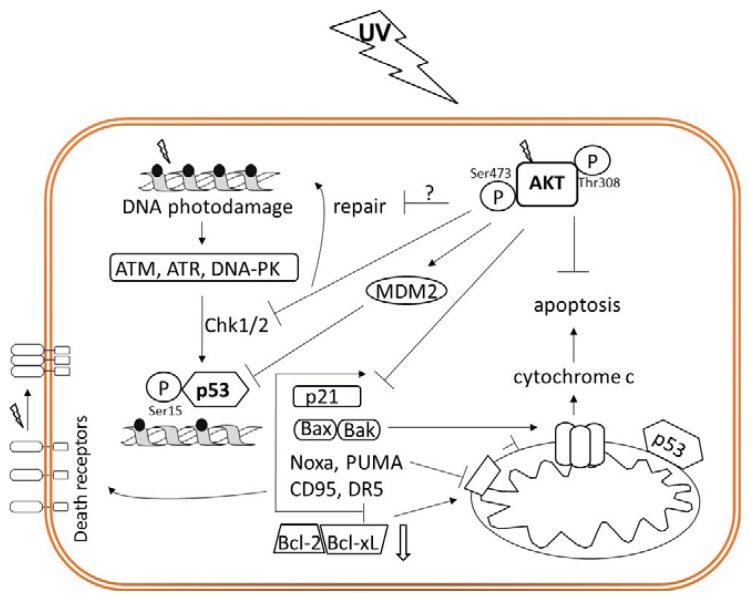
p53-induced cell cycle control and apoptosis. UV-induced DNA damage activates ATR, ATM and DNA-PK kinases, which via check point kinases Chk1/2 signal to activate p53 and DNA damage repair. Activated p53 transcriptionally regulates the cell cycle control protein p21 and several components of the pro-apoptotic pathway. Pro-apoptotic Bax, Bak, Noxa and PUMA proteins and additionally death receptors CD95 and DR5 become up-regulated while p53 trans-represses anti-apoptotic Bcl-2 and Bcl-x_L_. Moreover, p53 induces apoptosis by direct interaction with the mitochondrial membrane. UV-activated AKT inhibits p53 by activation of its regulator MDM2 and/or by inhibition of Chk1/2. Above that, by inhibition of Chk1/2 AKT may interfere with DNA damage repair and directly inhibit p21.

The pivotal role of p53 in the elimination of severely damaged cells is underlined by the clinical evidence that p53 mutations induced by UV were found in >90% of human SCC and about 50% of BCC [57]. Keratinocytes with mutated p53 are designated to repair rather than to apoptosis induction, which is depicted by decreased amounts of apoptotic cells in the epidermis (sunburn cells, SC) [58–60]. Since cellular repair mechanisms are often error-prone, harmful mutations accumulate, and in concert with dysfunctional p53, cause an increased risk for malignant transformation. [58,60,61]. The activity of p53 is tightly controlled by its negative regulator MDM2. It prevents the transactivation capacity of p53 and/or designates it for proteasomal degradation. The activity of MDM2 again relies on AKT-dependent phosphorylation, providing another mechanism how AKT can prevent p53-mediated apoptosis [40,41]. Consequently, UV-induced AKT activation may contribute to malignant transformation in the skin (Figure 3). In turn, p53-induced apoptotic processes can commit AKT to cleavage by executioner caspases, thereby creating a feedback loop to protect cells from the adverse effects of AKT [40]. Taken together, an intense cross talk between pro-apoptotic p53 driven and anti-apoptotic AKT-mediated pathways seems to exist within the irradiated cells, while the balance between these pathways determines the fate of the cell.

## 5. AKT/mTOR Pathway Impedes UV-Induced Apoptosis

Experimental data in HaCaT cells bearing two heterozygous p53 mutations [62] implied p53-independent mechanisms to be also involved in UVB-induced cell death. Accordingly, ROS generated upon UV irradiation have been recognized to serve as additional initiators of the intrinsic apoptotic pathway directly causing mitochondria damage [63,64]. Moreover, UV radiation was shown to trigger cell death receptors (CD95/Fas; TRAIL; TNFR1) either by autocrine release of death ligands or directly, in a ligand-independent manner. Consequently, UV additionally induces the extrinsic apoptotic pathway [63,65].

Upon death receptor (CD95, TRAILR, TNFR) activation the extrinsic pathway is initiated by binding of the adapter protein FADD to the activated intracellular death domain (DD) and recruitment of the initiator procaspase-8 to form the death inducing signaling complex (DISC). In type I cells auto-activation of caspase-8 causes direct activation of downstream effector caspase-3 through proteolytic cleavage. In contrast, in type II cells the apoptotic signal generated at DISC is amplified via the intrinsic mitochondrial pathway [66,67]. Here, caspase-8 truncates pro-apoptotic Bid to translocate to the mitochondrial outer membrane where it interacts with Bcl-2 or Bcl-x_L_ to initiate the oligomerization of Bax and Bak. In this context, AKT-dependent inhibition of p53 and subsequently decreased levels of Bax and Bak can be expected to turn down the mitochondrial amplification loop. In addition, it is presumed that AKT may inhibit UV-induced apoptotic death receptor pathway by activation of NFκB and its targets including cellular inhibitor of apoptosis proteins (c-IAPs), caspase-8 inhibitory protein (FLIP), and TNF-R-associated factors 1/2 (TRAF1/2) [68,69]. Surprisingly, activation of NFκB by interleukin-1 (IL-1) was shown to enhance the apoptotic response to UVB (300 J/m^2^) in epithelial cells and keratinocytes [70]. The mechanism was associated with inhibition of negative feedback regulation of NFκB. Consequently, prolonged NFκB activation causes accelerated TNFα production followed by autocrine TNF-R1 activation. In parallel, NFκB-dependent repression of genes encoding inhibitors of apoptosis FLIP and cIAP as well as of TNFR adaptor proteins TRAF1, TRAF2 was responsible for shifting the balance from anti- to pro-apoptotic signal transduction at the TNF-R1 [71–73]. This phenomenon was also reported to play an important role in epithelial cells in response to base modifications-inducing agents UVA and cisplatin. In contrast, it remained absent in cells where etoposide, doxorubicin or γ-radiation induced DSBs, indicating that the type of DNA lesion modulates the NFκB-dependent responses [74]. In fact, following UV-irradiation IL-1 and TNFα are produced in high amounts by epidermal keratinocytes, implicating this mechanism to significantly modulate the physiological responses to UVB *in vivo* [72,75]. At the single cell level, activation of AKT in response to UVB seems to precede a weak and delayed activation of NFκB [24,72], implying possible uncoupling of these pathways during UV responses. Still, the potent anti-apoptotic activity of AKT is strongly underlined by the fact that inhibition of AKT caused a threeto four-fold increase of UV-induced apoptosis (500 J/m^2^) in human normal keratinocytes, becoming evident by enhanced caspases-9 and -8 processing and cytochrome c release [24]. Similarly, a sub-lethal dose of the AKT inhibitor perifosine effectively enhanced UVB-induced loss of viability of primary keratinocytes and fibroblasts two- to fivefold at 150 J/m^2^ and 200 J/m^2^. Loss of viability ascribed to apoptotic cell death comprised decrease of cellular Bcl-2 and Bcl-x_L_ suggesting p53-dependent pathways to be involved. Additionally, knockdown of mTOR or Rictor implied AKT-mediated keratinocyte survival to depend on mTOR signaling [76]. This was analyzed in a series of elegant experiments by Carr *et al*. [77] who described the functional distinction of mTOR complexes (mTORC1 and mTORC2) in murine epidermis during UVB-induced apoptosis. Unlike the rapamycin-induced inhibition of mTORC1, keratinocytes were sensitized to high-dose (500 J/m^2^) UVB only when mTORC2 activity was down-regulated. Conversely, rapamycin effectively reduced G1 to S cell cycle progression in synchronized HaCaT cells and, similarly to the deletion of mTOR, blocked the hyper-proliferation of keratinocytes that occurs upon sub-lethal UVB doses *in vivo*.

Thus, hyper-activation of the AKT/mTOR pathway in response to UV radiation is associated with death resistance through inhibition of apoptosis and with mTORC1-driven hyper-proliferation. This implies, that the AKT/mTOR pathway allows damaged keratinocytes to survive and proliferate under hazardous conditions and thereby it contributes to photocarcinogenesis.

## 6. Implication of the AKT/mTOR Pathway in Photocarcinogenesis

Analyzing the mutational spectrum found in SCC, BCC and melanoma, respectively, AKT/mTOR signaling at a first glance does not seem to play a prevalent role in skin carcinogenesis. Recently, however, the phosphorylation status of mTOR signaling components was assessed in immunohistochemical and protein microarray analyses of human epidermal tumors in comparison to normal tissue [78,79]. Enhanced phosphorylation of mTOR, 4E-BP1 or S6K and its target protein S6 correlated with elevated expression of Cdk2, a cyclin-dependent kinase playing a pivotal role in G1 to S cell cycle transition. These data strongly indicate that signaling downstream from mTOR forces cell cycle progression, thereby implying mTOR to play a crucial role in tumor promotion [78]. The link to UV irradiation however cannot be established. Similarly, mutations of *BRAF* and *NRAS* found with high frequency in melanoma seem not to result from the UV irradiation. Conversely, mutations of *PTEN* detected in some primary melanomas were shown to bear an UV fingerprint [3], which together with the fact that UV possesses a capacity to activate AKT/mTOR cascade, substantiates the involvement of AKT and mTOR in photocarcinogenesis.

Despite the fact that a limited amount of data exists concerning the direct impact of UV radiation on AKT pathways in melanocytes, the activity of AKT and ERK has been shown to diminish skin pigmentation. Due to the resulting lack of keratinocyte pigmentation, AKT and ERK may indirectly contribute to increased sensitivity towards UV radiation [80]. While the data on direct UV-triggered activation of ERK in human keratinocytes is inconsistent [23,35,81] the impact of UV irradiation on PI3K/AKT/mTOR activation *in vitro* and *in vivo* as well as in murine skin is well established [21–25]. Indeed both, UVA (20/40 kJ/m^2^) and UVB (200/400 J/m^2^) at sub-lethal as well as apoptotic doses were shown to activate mTOR and its target S6K in a phosphorylation-dependent manner [81]. Along this line, activation of the epidermal growth factor receptor 2 (Erbb2) was shown to suppress cell cycle arrest by PI3K/AKT-dependent inhibitory phosphorylation of Chk1 (Ser280) and by maintenance of the cell cycle regulator Cdc25a in UVB/UVA irradiated murine skin. On the contrary, inhibition of UV-induced Erbb2 activation resulted in milder epidermal hyperplasia and S-phase accumulation of cells [36]. Similarly, human HaCaT cells and normal human keratinocytes exposed to sub-lethal UVB or UVA radiation did not arrest but progressed through G1/S in an EGFRand AKT-dependent manner [25,35] and counteracted the G2/M checkpoint by conveying an inhibitory phosphorylation of Chk1 (Ser280) [34].

Taken together, hyper-activation of the AKT/mTOR pathway that occurs at a wide range of UVA and UVB doses supports epidermal tumor promotion by enforcing cell cycle transition and accelerated proliferation. According to Carr *et al*. cell survival *versus* proliferation diverge on mTOR complexes. Therefore, the inhibition of either both of the mTOR complexes or mTORC1 concomitantly with AKT, may represent a potential strategy to prevent photocarcinogenesis [77]. This concomitant inhibition is of particular importance, since prolonged treatment with rapamycin and its analogues was shown to induce a feedback to activate AKT [82–84]. However, such conclusion has to be stated with caution, since mTOR is also an important negative regulator of autophagy [85]. When induced by UV-stress, this process can trigger the escape from apoptotic clearance and promote long-term survival of precancerous cells.

## 7. Alternative Roles of p53 and AKT/mTOR Pathways in UV Responses: Autophagy

Autophagy is a highly conserved catabolic process of “self-eating” aimed to remove long-lived or damaged proteins and organelles, and for recycling of cytoplasmic contents. This adaptive response enables the cells to maintain homeostasis and to survive starvation stress. Thus, under physiological conditions induction of autophagy may suppress tumorigenesis. In established tumors however, autophagy facilitates cancer cells to survive either its own exhaustive metabolic turnover or therapeutic intervention [86–90]. Depending on the physiological context and stress stimuli autophagy can play a dual role: either it enables cells to escape from cell death or it contributes to cell death, called type II cell death or autophagic cell death (ACD) [87,91].

Autophagy induced by nutrient starvation has been most extensively studied, however recently a number of other stress factors such as UV radiation, DNA damage, ROS formation, hypoxia, and unfolded protein responses have been recognized to activate this process. In this context, autophagy is believed to predominantly rescue cells from stress-induced cell death and therefore can foster the survival of altered ones [92–94].

At the molecular level, autophagy is controlled by highly conserved autophagy-related *ATG* genes crucial for initiation, formation and maturation of autophagosomes whose cargo becomes degraded by hydrolases upon fusion with lysosomes [89,93,95]. Initiation of autophagy is regulated by ULK1/2 kinases (ATG1), which in a complex with the ATG13–FIP200 mediate inhibition of mTORC1. Reciprocally, ULK1/2 becomes activated upon mTORC1 inhibition for instance during starvation [85,88,93,96]. Next, Bcl-2-interacting protein-1 (Beclin-1; ATG6) in a complex with PI3KC3 orchestrates initial steps in autophagosome formation. This process requires interaction with Beclin-1 essential activator UVRAG (UV radiation resistance associated gene), initially described as a putative tumor suppressor that complements UV sensitivity [97]. Importantly, reduced levels of Beclin-1 attenuate UVRAG-induced autophagosome formation and *vice versa* [98]. Autophagosomal elongation is then associated with processing of the cytoplasmic microtubule associated protein 1 light chain 3 β (LC3β), which when truncated and lipidized to its LC3-II isoform integrates into the autophagosomal membrane, and serves as an autophagy-specific marker [99].

**Figure 4:**
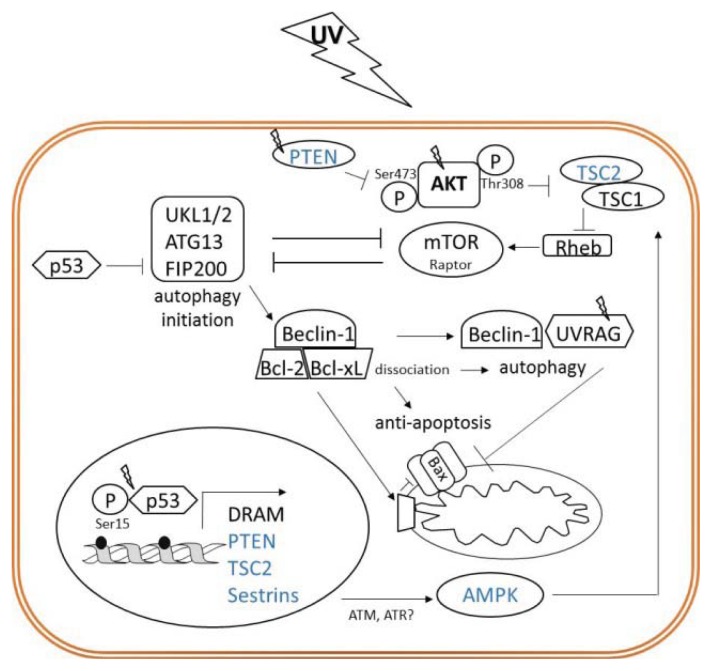
The interplay between p53 and AKT/mTOR in regulating autophagy. UV stress-induced p53 regulates the expression of damage-regulated autophagy modulator (DRAM) and factors mediating inhibition of AKT/mTOR (in blue). Inhibition of mTOR results in activation of ULK1/2–ATG13–Fip200 autophagy initiating complex. Consequently, dissociation of Beclin-1/Bcl complex enables Beclin-1 to interact with UVRAG activating next steps in the process of autophagy. Bcl proteins can now exert their anti-apoptotic function by counteracting Bak or Bax. The pro-apoptotic function of Bax can additionally be inhibited by up-regulated UVRAG.

With regard to UV radiation, it is sensible to conclude that autophagy would contribute to maintenance of UV-damaged cells because one of its major regulators UVRAG was named to reflect its role in providing resistance against UV [98]. Indeed, inhibition of UV-induced autophagy has been shown to reduce cell viability and enhance apoptosis [92,100,101]. The fact, that UV may induce autophagy seems to be contradictory to UV-mediated activation of the AKT/mTOR pathway. Yet, induction of autophagy is likely to be regulated by UV-induced p53, described to act as a bidirectional regulator of autophagy [102,103]. While under stress conditions transcriptional activity of p53 acts in favor of autophagy, a cytoplasmic pool of p53 suppresses autophagy by a not yet fully understood mechanism [85]. The only essential autophagy related protein that is known to interact with p53 is FIP200, a multifunctional protein that is present in the autophagy activating ULK1/2 complex [93]. The pro-autophagic nuclear activity of p53 comprises transactivation of the damage-regulated autophagy modulator DRAM and negative regulators of AKT/mTOR pathway (Figure 4) [102]. Transactivation of *PTEN* negatively regulates AKT activity together with AKT-dependent activation of mTORC1 [94]. Further, inhibition of anti-autophagic mTORC1 can be enhanced by p53-mediated transactivation of *TSC2* gene. TSC2 together with TSC1 blocks mTORC1 and its inhibitory function on autophagy [102]. Furthermore, in response to DNA damage and oxidative stress, p53 induces expression of stress-induced proteins called sestrins functioning as antioxidants and inhibitors of mTORC1. Sestrins activate AMPK which positively regulates autophagy related targets, the TSC complex, ULK1 and p53 itself [94,102]. In addition to growth factor receptor-mediated activation of AMPK [37], repression of mTOR signaling through the AMPK and TSC complex has been linked to ATM, which for the first time was indicated to act via a novel cytoplasmic signaling pathway [104]. This novel mechanism seems to be independent of its conserved nuclear function in genotoxic stress response, but it is rapidly induced to suppress mTORC1 signaling by oxidative stress. Based on the homology of ATM and ATR in their catalytic domains [105], this mechanism might be speculated to be involved in the cytoprotective autophagy, which appeared in an ATR-dependent manner at early stages of UV-induced apoptosis [92]. Conclusively, UV can initiate autophagy through negative regulation of mTOR by p53 in response to DNA damage and in response to ROS by AMPK activated downstream of growth factor receptors or through ATM.

Authophagy meets apoptosis at an interplay between ATG and anti- as well as pro-apoptotic proteins. It is postulated that these two pathways converge at Beclin-1, which via its BH-3 domain interacts with the anti-apoptotic proteins Bcl-2, Bcl-x_L_ or Bcl-w [106,107]. Indeed, autophagy promoting Beclin-1-PI3KC3 complex is suppressed by Bcl proteins implying that, additionally to their anti-apoptotic function, Bcl proteins also act as inhibitors of autophagy. On the other hand, it suggests that the sequestration of Bcl proteins in the Beclin-1-PI3KC3 complex may sensitize cells to apoptosis [98,106]. Conversely, as shown by interaction of Beclin-1 with Bad, pro-apoptotic BH3-only proteins or BH3 mimetics can induce autophagy by competitively disrupting the interaction of Beclin-1 with Bcl-2/Bcl-x_L_ [107]. Although BH-3 domain containing Beclin-1 was not supposed to induce apoptosis, Beclin-1 loses its potential to induce autophagy when cleaved by caspases during execution of apoptosis. Subsequently, truncated Beclin-1 even contributes to apoptosis by direct interaction with the mitochondrial membrane causing release of cytochrome c. This indicates that once initiated, the apoptotic process inhibits autophagy by generating pro-apoptotic Beclin-1 fragments being unable to induce autophagy [108]. An active pro-apoptotic function of cleaved Beclin-1 is in agreement with the reported lack of enhanced apoptotic responses to UV irradiation in Beclin-1 deficient ES cells [109]. This suggests that UV-induced apoptosis antagonizes autophagy at the level of Beclin-1. However, another player namely UVRAG, found to be up-regulated upon genotoxic stress, exhibits an anti-apoptotic activity additionally to its role in promoting autophagy. In tumor cells exposed to chemotherapy or UV radiation, up-regulated UVRAG exerted its anti-apoptotic function by preventing the translocation of Bax to the mitochondria [101]. Consequently, knockdown or down-regulation of UVRAG has been shown to reduce UV-induced autophagy in favor of apoptosis [100,101]. According to this data, the decrease in UVRAG expression is pro-apoptotic by two independent ways. One proposed mechanism of negative UVRAG regulation has been shown to rely on AKT in a kinase-independent manner. Overexpression of AKT in HEK293 and breast cancer cells inhibited UV-induced autophagy and reduced autophagy-associated proliferation. Thus, AKT has been postulated to counteract autophagy not only due to activation of mTOR, but also by down-regulation of UVRAG. However, AKT over-expression attenuated UV-induced apoptosis, indicating its prevalent role in inhibiting apoptosis over pro-apoptotic inhibition of autophagy in these cells [100]. Another way to induce autophagy instead of apoptosis in response to UV was documented in JB6 murine epidermal cells. The mechanism was proposed to rely on the UVB-mediated inhibition of glycogen synthase kinase 3 β (GSK3β). UVB-induced (1000 J/m^2^) appearance of the autophagy marker LC3-II was decreased by over-expression of wild-type or constitutively active GSK3β and was accompanied by increased UVB-induced cell death [110]. Keeping in mind that UVB and UVA, both, potently activate AKT, which downstream inhibits GSK3β [111], plus the fact that AKT inhibits autophagy by mTOR activation and possibly by down-regulation of UVRAG, the data appear to be disparate. Thus, the exact role of AKT in inhibition of autophagy *versus* inhibition of apoptosis in UV-associated responses remains functionally insufficiently explored. So far, the available data suggest that autophagy relies on a reciprocal regulation of p53 and AKT/mTOR to counteract UV-induced apoptosis. It might be surprising, that the process of autophagy is generally negatively regulated by AKT/mTOR pathway but driven by p53, as it is the fact for apoptosis. This indicates that stress responses, although utilizing common signal molecules, are very complex and only a minor change in molecular equilibrium can have serious physiological consequences.

Recently, another interesting aspect of autophagy has been indicated to influence skin physiology, namely the regulation of melanosome degradation in keratinocytes. In a study on the regulation of skin pigmentation among different ethnic groups, Murase *et al*. showed that melanosome accumulation in keratinocytes is associated with knockdown of essential autophagy-related genes. Additionally, the melanin levels in human skin samples cultured *ex vivo* and in human skin substitutes *in vitro* were substantially diminished by activators of autophagy and enhanced by its inhibitors [112]. In this context, autophagy-mediated melanosome degradation might contribute to UV-induced damage of keratinocyte *in vivo*. In this particular aspect, autophagic clearance of melanosomes could either contribute to DNA damage-induced apoptosis or malignant transformation, depending on the individual status of p53 and AKT activity.

## 8. p53-dependent DNA Damage Responses and Oncogenic Pathways Induce Senescence

DNA damage-induced ATM and p53 both being involved in apoptosis and autophagy induction, respectively, have also been described as key players in induction of senescence. Senescence describes an irreversible cell cycle arrest, and in contrast to quiescence appears only in cells maintaining active metabolism or persistent oncogenic signaling, such as mediated by the best studied oncoprotein Ras. Here, oncogenic hyper-proliferation resulting in high DNA replication rates activates DNA damage responses [113]. This mechanism seems to be acquired in order to cope with the oncogenic pathways, which prevent apoptosis and/or enforce proliferation. Thus, senescence represents a physiological anti-tumor response [113–116]. Accordingly, melanocytes harboring oncogenic *BRAF* mutations can be protected from malignant transformation by oncogene-induced senescence. Inactivation of p53 in BRAF(V600E) melanocytes enables their survival and proliferation leading to immortalization and transformation both *in vitro* and *in vivo* [115]. Hence, functional p53 is fundamental for oncogene-induced senescence to prevent malignant transformation [115,117]. This is believed to explain why only a minority of benign melanocytic nevi frequently expressing BRAF(V600E) finally progress to malignant melanoma [115].

At the molecular level, senescence is triggered by intracellular accumulation of oxidative damage induced by ROS, and depends on cell cycle arrest mediated by the p53 targets p21 and E2F combined in a complex signaling network with cyclin-dependent kinase p16 and retinoblastoma Rb [117,118]. In UVB-irradiated keratinocytes increased ROS and maintenance of p21 followed by induction of senescence has been ascribed to the activity of insulin-like growth factor 1 receptor (IGF-1R). Since IGF-1R similar to other RTKs like EGFR or Erbb2 potently induces AKT signaling pathway [119], it might indicate its involvement in the induction of senescence. In fact, senescence induction as a consequence of UVB-mediated ROS generation in human keratinocytes, has been linked to PI3K/AKT-dependent pathways regulating activation of NADPH oxidases. Activation of PTEN resulted in inhibition of ROS and diminished occurrence of senescence *in vitro* and in murine skin *in vivo*. [120]. Following this line, due to a stimulatory role in growth promoting pathways and anabolic processes mTOR has been recognized to play a key role in mediating senescence. In contrast, lack of mTOR activity (e.g., by starvation of rapamycin treatment) causes quiescence in arrested cells [115,117]. Thus, p53-dependent inhibition of the mTOR pathway described in the former section to be pro-autophagic is responsible for the paradoxical function of p53 in the suppression of senescence [121] and may possibly explain a p53-dependent cell cycle re-entry of senescent cells observed under certain conditions [122]. Although there is little definitive experimental evidence on UV-induced senescence, the activation of AKT/mTOR pathway in cells bearing functional p53 implicates senescence to occur in irradiated cells to protect them against malignant transformation.

## 9. UV Does Not Act Alone: Impact of Heat Shock and Infrared on UV Response

During tanning or medical applications, exposure of the skin to UVB, and particularly UVA, is accompanied by a local increase in temperature. At the molecular level, hyperthermia induces denaturation of intracellular enzymes and other proteins. Although a heat shock up to 45 °C does not induce DNA damage *per se*, Takahashi *et al*. [123] indicated that heat treatment contributes to DNA damage in form of DSBs via dysfunction of heat-labile repair proteins, e.g., DNA polymerase β. Recently, Krawczyk *et al*. [124] revealed that heat anticipates DSB-repair by heat-induced degradation of BRCA2, an important factor involved in DSBs recognition and recruitment of HRR enzymes. In principle, the loss of HRR is generally more toxic than mutagenic [125], thus it may be expected that a concomitant exposure of cells to UV and to increased temperatures will result in forced death of cells harbouring DSBs induced secondarily to photoproducts [14].

However, heat treatment may also induce cytoprotective responses. One of the best known mechanisms involve the induction/activation of heat shock family of proteins (HSP), particularly HSP27 and HSP70, to protect intracellular proteins from denaturation, damage and degradation [126]. Moreover, HSP70 has an established role in preventing apoptosis as a negative regulator of mitochondrial dysfunction in response to different stress stimuli including UV. HSP70 prevents mitochondrial membrane permeabilization through inhibition of Bax, it interacts with AIF and Apaf-1 and consequently prevents maturation of effector caspases [127–130]. Thus, upon either UVA or UVB irradiation the heat shock response in terms of HSP induction may have a significant impact on cellular responses. In fact, elevated HSP expression has been identified in upper epidermal cells and HSP70 was shown to protect against ROS- or UVB-induced cell death in keratinocytes [131,132]. Following this line, mutated heat shock factor (HSF1) incapable of conveying HSP70 transactivation failed to prevent UV-induced apoptosis in mouse embryonic fibroblasts [133]. Thus, HSP70 seems to represent another pro-survival factor in irradiated potentially cancerous skin cells.

In addition, HSP70 may indirectly affect photocarcinogenesis as it was reported to accelerate depigmentation in a mouse model of vitiligo [134] and to suppress production of melanin [135]. Just like autophagy-mediated melanosome degradation mentioned before [112], the decrease in melanin production may render epidermal keratinocytes more susceptible to UV-induced damage.

Besides induction of cytoprotective HSPs, heat may also induce the activation of AKT [136,137]. AKT activity has been shown to be involved in regulating HSP70 induction [138] which putatively may strengthen its anti-apoptotic and pro-proliferative effect during UV responses. Summing up the versatile tasks fulfilled by AKT in UV responses it can be speculated that heat changes the relation between AKT and p53 signaling and may additionally modulate autophagic and senescence-inducing mechanisms. However, there is insufficient experimental data to paint a big picture.

Concomitantly with increased exposure to UV light human skin is increasingly exposed to infrared (IR) radiation, which contributes to about 53% of solar radiation and finds wide application in wellness facilities. Importantly, IR-A (700–1400 nm) penetrates deep into the dermis to modulate UV response. IR has been reported to diminish UVB-induced apoptosis in mouse keratinocytes *in vitro* and to limit sunburn cells in the epidermis of irradiated mice. Although IR can intuitively be associated with increased temperatures, it has been shown to signal without the induction of HSP70. The decrease in apoptotic responses to UVB coincided with an increase in DNA repair most probably by enhancement of the NER. Furthermore, IR also reversed UVB-induced down-regulation of anti-apoptotic FLIP and Bcl-x_L_, and mediated up-regulation of Bax. Thus, IR besides its thermal effects negatively modulates all known apoptotic pathways triggered by UVB, including the one induced by DNA damage, and both extrinsic and intrinsic pathways [139]. A photocarcinogenetic study using mouse models revealed that although IR delayed appearance of UVB-induced skin cancer, it was causative for enhanced aggressiveness of developed sarcomas and epithelial tumors [140].

## 10. Conclusions

**Figure 5:**
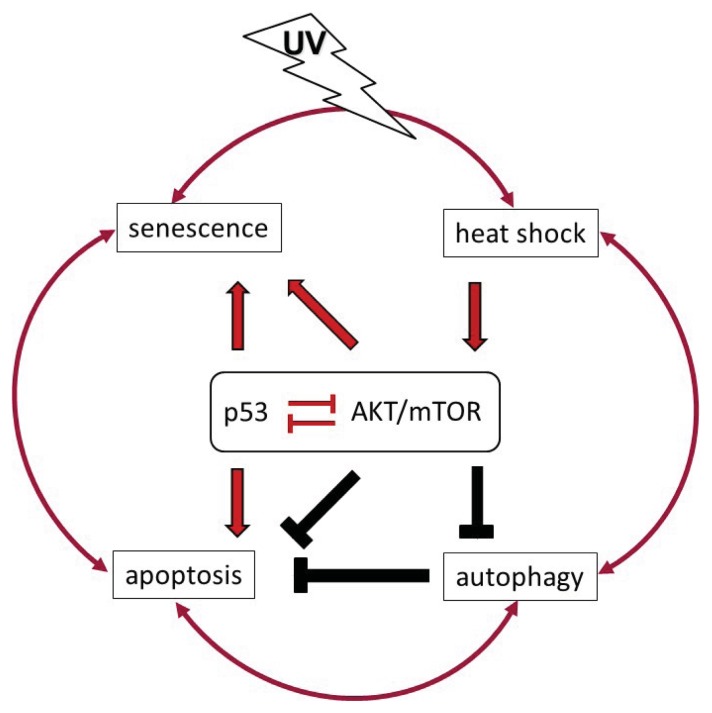
The balance between p53 and AKT/mTOR determines the fate of the UV-irradiated cells. p53 and AKT/mTOR mutually inhibit each other in a tightly regulated cross talk, that influences cellular responses at different levels. UV-induced p53-dependent apoptosis can be inhibited by UV- or heat-activated AKT/mTOR pathway. *Vice versa*, UV-activated p53 can inhibit AKT/mTOR thereby counteracting its anti-apoptotic function but contributing to induction of autophagy. However, UV-induced autophagy counteracts UV-induced apoptosis at least to certain extends. Under slightly different physiological conditions, concomitant activation of p53 and AKT/mTOR can drive cells into senescence and thereby mediate an anti-tumor response. Thus, a very complex regulation of cell death and survival pathways exists, that controls malignant transformation of skin cells.

UVB and UVA radiation are broad spectrum hazards that affect exposed skin cells by induction of DNA damage, ROS formation and receptor activation, respectively. UV radiation triggers death as well as survival pathways, which balance the fate of the cell. In order to address the question which physiological response will result from UV exposure *in vivo* multiple aspects have to be taken into account. These include dose, frequency and duration of UV exposure, accompanying exposure to other types of radiation, generation of heat and influence of neighboring cells. At the molecular basis, the tumor suppressor p53 is responsible for sensing the intensity of DNA damage to induce cell cycle arrest to either guaranty DNA repair or to commit the cell to apoptotic death in favor of the surrounding tissue. However, aside to this black and white scenario a complex cross talk exists between p53-mediated cell death and cell survival pathways. Amongst the pro-survival signaling pathways, the AKT/mTOR branch appears to play a major role in tuning p53 activity. Consequently, not only antagonism of cell death but also a shift to other physiologically relevant long term conditions may be provoked by a sensitive interplay between p53 and AKT/mTOR (Figure 5). Especially induction of senescence or autophagy either terminates cell proliferation in pre-cancerous state or allows long term survival under physiologic stress conditions. In both cases, changes of the molecular p53-AKT/mTOR balance may again commit the cells to apoptotic cell death or may push them back into the cell cycle with a potential for malignant transformation. Transitions between the listed conditions are smooth and may sometimes rely on the concentration of a single or very few proteins within the cell.

Taken together, UV radiation triggers a huge sensitive and complex signaling network that provides a platform for numerous and variable cellular responses, that need to be taken into account when discussing e.g., formulation of sunscreens. Furthermore, many features of UV-induced cellular responses also translate to conditions that are relevant for phototherapy. Consequently, knowing more about the molecular signalling network appears to be essential not only for the therapy but also for the prevention of skin cancer.
